# Widespread Interest in Eugenics

**Published:** 1912-12

**Authors:** 


### Widespread Interest in Eugenics.

To change the mores (folk lore) of a people is a difficult task, and it is rarely ever accomplished in several generations. But so long as a race advances it will change its accustomed ideals and old-seated prejudices. And the higher the civilization, the more transitional are these changes. But it remains for eugenics to outdo all other innovations. Never has there been an idea advanced, for the welfare of society, that has taken such complete hold on the people. It has been but twenty-five years since Galton first suggested the idea that the race could be improved, and the last five years has seen a general adaptation of his teaching by educators and laymen alike. Indeed, the principles of eugenics form the basis of all human endeavor, and the social reforms of the future will be developed from this standpoint. England has just held a National Congress of Eugenics, and eugenic societies are being organized all over the country. It formed a conspicuous part in the recent Congress of Hygiene and Dermography held at Washington. It is the purpose of this department to keep to the front of this movement for the instruction and benefit of the "RED BACK'S" rapidly growing family.

